# Moped vs. truck: case of traumatic tricuspid valve rupture and successful repair

**DOI:** 10.1093/ehjcr/ytaf234

**Published:** 2025-05-12

**Authors:** Michael Hii, Gareth Crouch, Kaylene Gluyas, Majo X Joseph

**Affiliations:** Department of Cardiology, Flinders Medical Centre, Flinders Drive, Bedford Park, Adelaide 5042, Australia; Adelaide Medical School, University of Adelaide, North Terrace, Adelaide 5000, Australia; Department of Cardiology, Flinders Medical Centre, Flinders Drive, Bedford Park, Adelaide 5042, Australia; Department of Cardiology, Flinders Medical Centre, Flinders Drive, Bedford Park, Adelaide 5042, Australia; Department of Cardiology, Flinders Medical Centre, Flinders Drive, Bedford Park, Adelaide 5042, Australia; College of Medicine and Public Health, Flinders University, Sturt Road, Bedford Park, Adelaide 5042, Australia

## Summary

Traumatic tricuspid valve injury is a rare but well-described result of trauma comprising 0.02% of injuries.^1^ Mechanisms relate to transient extreme rise in intra-cardiac pressure or rapid deceleration injury.^2,3^ We describe a 19-year-old woman who suffered traumatic tricuspid valve injury during a motor vehicle accident. Massive tricuspid regurgitation was due to papillary muscle rupture, initially misdiagnosed as infective endocarditis due to positive blood cultures and vegetation-like appearance of the papillary muscle head. The patient underwent successful tricuspid valve repair with trivial residual tricuspid regurgitation. Understanding the mechanism of injury with a high degree of suspicion was crucial in rethinking the diagnosis and leading to a curative procedure.

## Case description

A 19-year-old woman suffered a motor vehicle accident with severe orthopaedic injuries whilst holidaying overseas. Initial echocardiography showed massive tricuspid regurgitation and a large 11 mm echogenicity attached to the anterior leaflet tip. The presentation was complicated by sepsis and blood cultures were positive for methicillin-resistant *Staphylococcus haemolyticus*; likely related to intravenous lines. Intravenous vancomycin was given for 4 weeks followed by oral linezolid for endocarditis.

**Figure 1:**
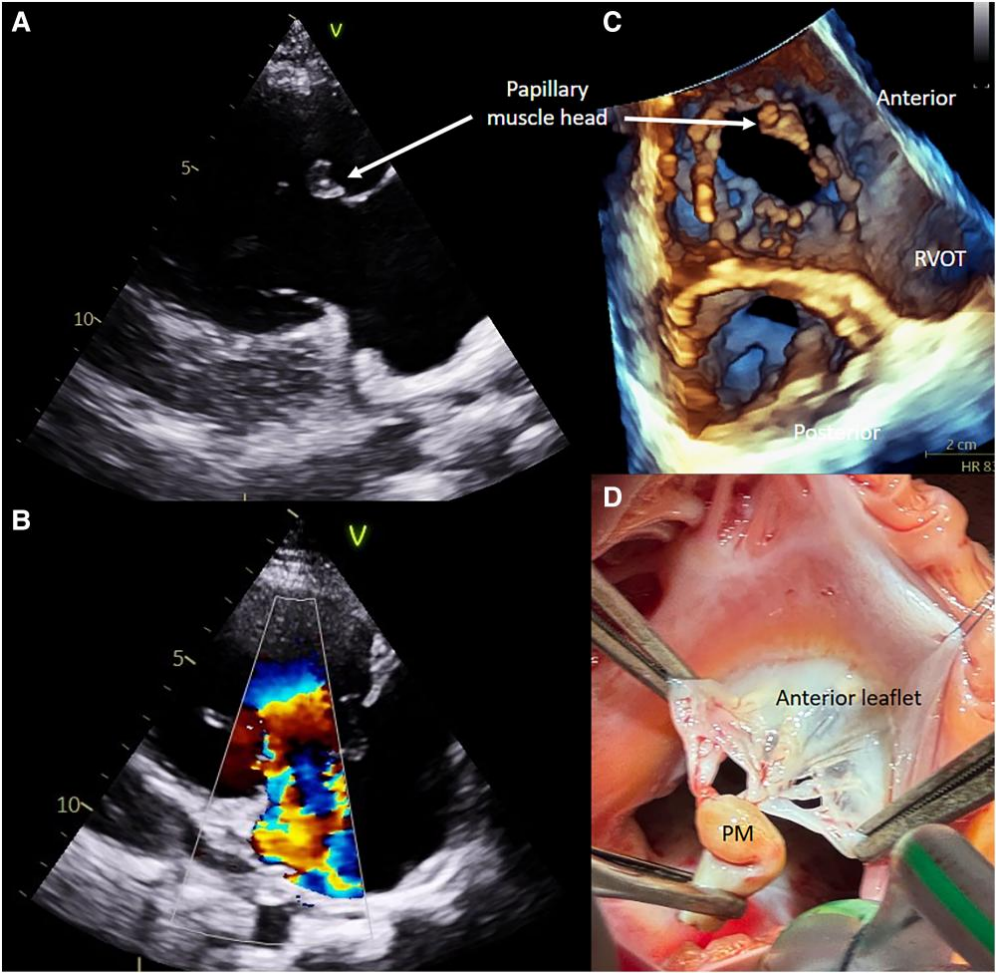
Echocardiographic and intraoperative views of traumatic tricuspid valve injury.

The patient was repatriated after initial orthopaedic treatment. Repeat 2D echocardiography was able to demonstrate the chordal attachment of the echogenicity and leaflet tip with its echodensity more consistent with myocardium (*Figure 1A*); there was persisting massive tricuspid regurgitation with flail anterior leaflet tip (*Figure 1B*). 3D transthoracic echocardiography localized the echogenicity to the expected location of the anterior papillary muscle on the en-face view (*Figure 1C*). Given the mechanism of injury, the echogenicity was concluded to be in-fact the severed anterior papillary muscle head. She was referred for tricuspid valve repair given severe right ventricular dilatation with moderately reduced systolic function and developing right heart failure requiring diuretic therapy.

Intraoperative examination showed papillary muscle rupture with flail anterior tricuspid valve leaflet (*Figure 1D*). Plication, closure of anterior leaflet cleft, and resection of the ruptured papillary muscle were performed; 28 mm Edwards tricuspid annuloplasty ring was implanted. Surgical pathology specimens were negative for endocarditis.

The patient recovered well and diuretic therapy was ceased at follow-up. Echocardiography showed trivial tricuspid regurgitation and only mild right ventricular systolic dysfunction at 6 months post-op.

## Supplementary Material

ytaf234_Supplementary_Data

## Data Availability

The data underlying this article will be shared on reasonable request to the corresponding author.

## Notes

**Consent:** Informed consent was obtained from the patient for publication.

**Funding:** No funding obtained.
